# MR imaging of the magnetic fields induced by injected currents can guide improvements of individualized head volume conductor models

**DOI:** 10.1162/imag_a_00176

**Published:** 2024-05-20

**Authors:** Fróði Gregersen, Hasan H. Eroğlu, Cihan Göksu, Oula Puonti, Zhentao Zuo, Axel Thielscher, Lars G. Hanson

**Affiliations:** Section for Magnetic Resonance, DTU Health Tech, Technical University of Denmark, Kgs Lyngby, Denmark; Danish Research Centre for Magnetic Resonance, Centre for Functional and Diagnostic Imaging and Research, Copenhagen University Hospital – Amager and Hvidovre, Copenhagen, Denmark; Sino-Danish College, University of Chinese Academy of Sciences, Beijing, China; High-Field Magnetic Resonance Center, Max-Planck-Institute for Biological Cybernetics, Tübingen, Germany; State Key Laboratory of Brain and Cognitive Science, Beijing MRI Center for Brain Research, Institute of Biophysics, Chinese Academy of Sciences, Beijing, China; Center for Excellence in Brain and Science and Intelligence Technology, Chinese Academy of Sciences, Beijing, China

**Keywords:** magnetic resonance current density imaging (MRCDI), echo planar imaging (EPI), computational head modeling, transcranial brain stimulation (TBS)

## Abstract

Volume conductor models of the human head are routinely used to estimate the induced electric fields in transcranial brain stimulation (TBS) and for source localization in electro- and magnetoencephalography (EEG and MEG). Magnetic resonance current density imaging (MRCDI) has the potential to act as a non-invasive method for dose control and model validation but requires very sensitive MRI acquisition approaches.

A double-echo echo-planar imaging (EPI) method is here introduced. It combines fast and sensitive imaging of the magnetic fields generated by the current flow of transcranial electric stimulation with increased robustness to physiological noise. For validation, noise floor measurements without injected currents were obtained in five subjects for an established multi-echo gradient-echo (MGRE) sequence and the new EPI method. In addition, data with current injection were acquired in each subject with a right-left (RL) and anterior-posterior (AP) electrode montage with both sequences to assess the accuracy of subject-specific detailed head models.

In line with previous findings, the noise floor measurements showed that the MGRE results suffered from spatial low-frequency noise patterns, which were mostly absent in the EPI data. A recently published approach optimizes the ohmic conductivities of subject-specific head models by minimizing the difference between simulated and measured current-induced magnetic fields. Here, simulations demonstrated that the MGRE noise patterns have a larger negative impact on the optimization results than the EPI noise. For the current injection measurements, a larger discrepancy was found for the RL electrode montage compared with the AP electrode montage consistently for all subjects. This discrepancy that remained in part also after optimization of the ohmic conductivities, was similar for the data of the two sequences and larger than the measurement noise, and thus demonstrates systematic biases in the volume conductor models.

We have shown that EPI-based MRCDI is superior to established techniques by mitigating the effects of previously reported spatial low-frequency noise in MRCDI if limited spatial resolution is acceptable. Additionally, the consistent inter-subject results indicate that MRCDI is capable of picking up inaccuracies in computational head models and will be useful to guide systematic improvements.

## 1 Introduction

Computational models of the human head are used to estimate current density (**J**) or electric field distributions in the brain and serve as important tools for dose stimulation and target optimization in transcranial brain stimulation as well as source localization in electro- and magnetoencephalography (Huang et al., 2019;Thielscher et al., 2015;Vorwerk et al., 2014). The accuracy of the head models has direct impact on the simulation results but is still uncertain even for detailed models based on individual structural MR images. For example, direct measurements using implanted electrodes in patients and non-human primates revealed on average reasonable fits between simulated and measured fields (Huang et al., 2017;Opitz et al., 2016,^2018^), but also strong deviations in several cases. In a similar vein, the ohmic tissue conductivities are not well known so far, contributing to the uncertainty of the simulated fields (Saturnino et al., 2019).

Recently, the possibility of non-invasively measuring the current-induced magnetic fields in the human brain by MRI has been demonstrated (Chauhan, Indahlastari, et al., 2018;Göksu et al., 2018,^2021^;Kasinadhuni et al., 2017). The technique termed magnetic resonance current density imaging (MRCDI) (Joy et al., 1989) measures phase perturbations of the MR images caused by the current-induced magnetic fields parallel to the scanners’ main magnetic fields with the aim of reconstructing the current density or conductivity (in which case the technique is referred to as MR electrical impedance tomography (MREIT)). Several current density and conductivity reconstruction approaches have been developed with different application foci. We use a recently suggested method that compares measured magnetic fields with simulated magnetic fields in subject-specific head models and optimizes the conductivities in the head models by minimizing the difference between measured and simulated fields (Eroğlu et al., 2021).

Although recent studies have demonstrated reliable detection of current-induced magnetic fields in the human brain (e.g.,Göksu et al., 2018,^2021^), spatial low-frequency noise was present in the magnetic field measurements, which compromises conductivity optimization. Additionally, only single brain-slice magnetic field measurements have so far been used in conductivity optimization due to the need for long scan times to increase the signal-to-noise ratio (SNR), especially in the low-frequency domain. All the previous in-vivo human brain studies used multi-echo gradient echo (MGRE) sequences.

In this study, we demonstrate the possibility of using double-echo echo planar imaging (EPI) to resolve the issue of low-frequency noise in the reconstructed magnetic field measurements. We compare an MGRE sequence with a time-matched 1-slice EPI sequence as well as a 5-slice EPI sequence (also time-matched, thus resulting in a lower SNR per slice).

We first validate the accuracy of the magnetic field measurements obtained with the EPI sequence with a wire loop experiment where the ground truth of the magnetic field inside the imaged volume is known. Secondly, we evaluate the influence of measurement noise from the 1-slice and 5-slice EPI and the MGRE measurements on the conductivity optimization in a simulation study. Finally, we compare the simulated and measured magnetic fields from five subjects with two-electrode montages using all three measurement approaches and perform conductivity optimization.

## 2 Methods

### 2.1 Data acquisitions

Five healthy volunteers were scanned in a 3T clinical MR scanner (MAGNETOM Prisma, Siemens Healthineers, Erlangen, Germany). Written informed consent was obtained from the participants prior to the scans and they were screened for contraindications to MRI and transcranial electric stimulation (TES). The study complied with the Helsinki Declaration on human experimentation and was approved by the Ethics Committee of the Capital Region of Denmark.

MRCDI data were acquired with both an optimized acquisition-weighted multi-gradient-echo (MGRE) sequence (Göksu et al., 2021) and with a single-shot double-echo EPI sequence provided by the Center for Magnetic Resonance Research (CMRR, Minneapolis, MN, USA). The imaging parameters for the MGRE sequence were T_R_= 80 ms, T_E_= 5.6, 14.4, 23.2, 32, 40.8, 49.6, 58.4, 67.2 ms, flip angle 30°, FOV 224 x 183 mm^2^, matrix size 176 x 144, and acquisition time 4:20 min. The same slice was scanned twice, resulting in a total acquisition time of 8:40 min. For more details on the acquisition-weighted MGRE sequence see (Göksu et al., 2021). The sequence diagram and injected current pattern for the double-echo EPI sequence is shown inFigure 1. The imaging parameters were T_R_= 120 ms, T_E_= 25.6, 63.48 ms, flip angle 30°, FOV 225 x 190 mm^2^, and matrix size 76 x 64. Optimal efficiency in MRCDI—a trade-off between the current-induced phase accumulation and${\text{T}}_{2}^{*}$—requires a relatively long encoding time and therefore also T_R_. SNR efficiency additionally requires a long readout time. However, severe geometrical distortions and${\text{T}}_{2}^{*}$signal loss occur for long EPI readout. Therefore, two k-space transversals were performed per excitation to reduce geometric distortion as well as to increase the robustness to low${\text{T}}_{2}^{*}$. B_0_field maps were also acquired prior to EPI to perform distortion correction on the measured magnetic field images (Jezzard & Balaban, 1995). The FUGUE toolbox in FSL 6.0.4 was used for the distortion correction.

**Fig. 1.**
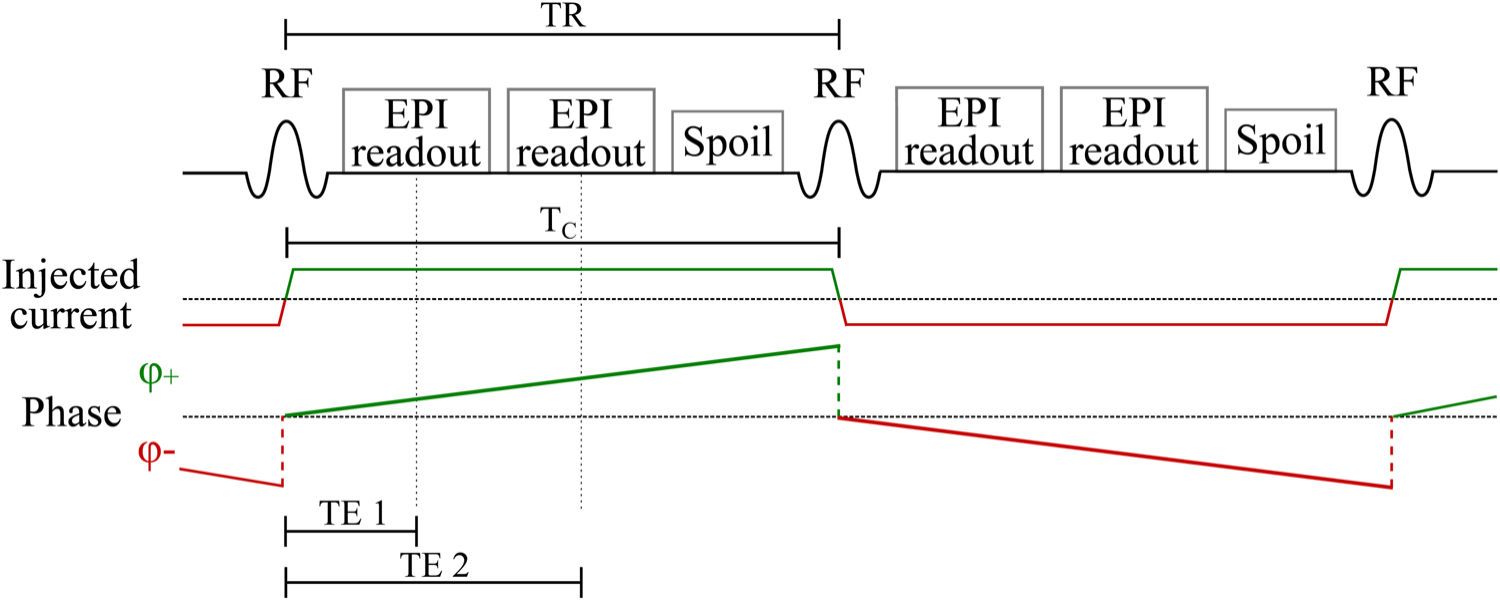
Diagram of the sequence and the current injection pattern. The current was injection in opposite directions for every other TR resulting in opposite phase accumulations. A single-shot double-echo EPI sequence with fly-back gradients was used. Lipid suppression is not shown.

A neurostimulator (DC-STIMULATOR MR, neurocare Group GmbH, München, Germany) and low-conductivity silicone rubber leads and surface electrodes (Gregersen et al., 2021) optimized for MRCDI were used for current injection. The neurostimulator was controlled by a signal generator reading trigger pulses from the MR scanner to synchronize the current injection with the MR pulse sequences. Alternating current injection directions were used in consecutive EPI measurements. The current-induced magnetic fields B_z,c_were then calculated from the difference in the phase images divided by the phase sensitivity of the sequence as B_z,c_= (φ^+^-φ^-^)/(2γT_E_), where γ is the gyromagnetic ratio of protons. B_z,c_estimates from separate echoes were combined based on the variance of B_z,c_for each echo calculated from the voxel-wise tSNR in the magnitude image.

One slice was repeatedly measured 4334 times (2167 B_z,c_images) resulting in an acquisition time of 8:40 min with the EPI sequence to time-match it to the MGRE acquisition. Additionally, 5 slices with 868 repeats (~1:44 min acquisition time and 434 B_z,c_images per slice) were acquired to obtain wider brain coverage while keeping the time matched to the single-slice EPI and the MGRE sequence. The slices were 1 cm apart. Each slice was fully acquired separately instead of performing interleaved acquisition to maintain a short TR and hence robustness to physiological noise. The FSL tool BET (Smith, 2002) was used to identify the brain on the EPI magnitude images and mask the B_z,c_measurements correspondingly.

A 3D structural image was acquired with the point-wise encoding time reduction with radial acquisition (PETRA) sequence (Grodzki et al., 2012) prior to MRCDI acquisition. The PETRA sequence is an ultra-short echo time sequence capable of imaging the silicone rubber lead wires. The leads were manually tracked, and the Biot-Savart law was used to calculate lead stray fields. These were subtracted from the MRCDI measurements (Göksu et al., 2019).

### 2.2 Computational head models and FEM simulation

Automatic head segmentation using T1- and T2-weighted MR images was performed to create individual volume conductor models of the subjects’ heads (Puonti et al., 2020). The Finite Element Method (FEM) implemented in SimNIBS 3.1 (Thielscher et al., 2015) was used to calculate the current density in the head for a given electrode montage and injection current strength. The magnetic flux density**B**was calculated from the current density using a fast Fourier transform-based method of solving the Biot-Savart integral (Yazdanian et al., 2020).

### 2.3 Optimization of conductivities

To optimize the tissue conductivities in the simulation, a constrained optimization problem was formulated with the aim of minimizing the root mean square (RMS) difference between the simulated (${\text{B}}_{\text{z,c}}^{\text{s}}$ ) and measured (${\text{B}}_{\text{z,c}}^{\text{m}}$ ) current-induced magnetic flux densities.

$$\begin{array}{l} \min\sigma \delta_{B_{z}}=\;\sqrt{\frac{1}{N}\sum_{i=1}^{N}{\left(B_{z,c}^{s}\left(\sigma ,i\right)-B_{z,c}^{m}\left(\sigma ,i\right)\right)}^{2}}\\ s.t.\;\;\;\;\;\sigma_{j}^{low}\leq \sigma_{j}\leq \sigma_{j}^{high}\;\;\;and\;\;\;\sigma_{WM}\leq \sigma_{GM\;}. \end{array}$$ (1)

Here,*i*labels the N voxels, and j labels each element in the tissue conductivity vectorσ. The tissue types used were white matter (WM), gray matter (GM), cortical cerebrospinal fluid (cCSF), scalp, and skull. The boundsσ^low^andσ^high^were [0.1 < σ_WM_< 0.4 S/m, 0.1 < σ_GM_< 0.6 S/m, 0.2 < σ_cCSF_< 1 S/m, 0.2 < σ_Scalp_< 1 S/m, 0.003 < σ_Skull_< 0.04 S/m]. Ventricular CSF (vCSF) and cCSF were separated into two different compartments. The conductivity of cCSF is expected to be lower than that of vCSF due to the presence of meningeal layers that are not included in the simulations.Jiang et al. (2020)suggested using a cCSF conductivity of 0.85 S/m to emulate the presence of meningeal layers. The vCSF was kept constant at 1.79 S/m, which is the conductivity of CSF at body temperature (Baumann et al., 1997). When only a constant current and no surface potential are enforced, all conductivities in the simulation can simultaneously be scaled with a constant value giving rise to the same current density and magnetic field. A constant vCSF acts as an anchor point for the other conductivities to keep them within biologically relevant conductivity ranges. Any bias in the assumed vCSF will therefore scale all conductivity estimates.

For a fast evaluation of${\text{B}}_{\text{z,c}}^{\text{s}}$for each new set of conductivities, a non-intrusive general polynomial chaos (gPC) expansion was used. For a detailed description of the optimization and the gPC, please refer toEroğlu et al. (2021)andSaturnino et al. (2019), respectively.

### 2.4 Experiment 1: Verifying the EPI sequence with a wire loop

The first experiment was performed to verify that the phase sensitivity to the current-induced magnetic field was as expected with the employed EPI sequence. A wire loop was placed around the subjects’ head to generate predictable magnetic fields in the measured brain volume. These depend only on the current flow in the cables and on their geometry. Two milliamphere currents were passed through the loop, as it has previously been shown that this current strength provides sufficiently strong fields in the brain to identify spurious field patterns in the measurements, for example, caused by insufficient spoiling (Göksu et al., 2021). The wire loop was imaged with the PETRA sequence. After manual tracking, the Biot-Savart law was used to estimate the field created in the imaged brain slices. The simulated field was subtracted from the measured field, and the residuals were used to assess the quality and accuracy of the measurements. This validation approach is feasible as the measured magnetic fields are fundamentally similar whether they are generated by unknown tissue currents or known cable currents that give rise to predictable fields.

One subject participated in this experiment. Five 3 mm thick slices with 1 cm distance between each slice were acquired. Each slice took 1:44 min to acquire resulting in a total acquisition time of 8:40 min.

### 2.5 Experiment 2: Optimization of conductivities with added noise floors

In this experiment, we tested the impact of measurement noise in MRCDI on the optimization of conductivities. This was done by simulating${\text{B}}_{\text{z,c}}^{\text{s}}$with a right-left electrode montage and with conductivities slightly different than the literature conductivities. Noise floor measurements (MRCDI measurements with zero current) were then added to the simulated${\text{B}}_{\text{z,c}}^{\text{s}}$before conductivity optimization. Note that both physiological and thermal noise is additive with respect to the current-induced magnetic field. Using this method, the ground truth of the conductivities is known, and therefore the error in the optimization can be calculated. However, when the electric potential at the electrodes is unknown, the conductivities can be scaled with a common constant and still give rise to the same current density and magnetic field in the brain. Therefore, the errors are more reliably estimated by taking the difference between the true (**J**^true^) and reconstructed (**J**^rec^) current densities, rather than the estimated conductivity values. The error is defined as the relative root mean square error of the current densities

$$\delta_{\text{J}}=\sqrt{\frac{\sum_{i=1}^{N}{\left|{\text{J}}^{rec}\left(i\right)-{\text{J}}^{true}\left(i\right)\right|}^{2}}{\sum_{i=1}^{N}{\left|{\text{J}}^{true}\left(i\right)\right|}^{2}}}\times 100\%,$$ (2)

where*i*labels the voxels. The conductivities used to create${\text{B}}_{\text{z,c}}^{\text{s}}$were [σ_WM_= 0.11 S/m, σ_GM_= 0.23 S/m, σ_cCSF_= 0.9 S/m, σ_Scalp_= 0.3 S/m, σ_Skull_= 0.012 S/m], while the initial conductivities in the optimization were set to the literature values used in SimNIBS [σ_WM_= 0.126 S/m, σ_GM_= 0.275 S/m, σ_cCSF_= 0.8 S/m, σ_Scalp_= 0.465 S/m, σ_Skull_= 0.01 S/m], except for the CSF since it was separated into cortical and ventricular CSF in this study. These conductivities will still be referred to as literature conductivities. Please note that, as we were solely interested in the impact of the different noise patterns on the optimization results, we could choose similar initial and ground-truth values for the conductivities here to facilitate a fast convergence of the algorithm.

The conductivity optimization was performed with the resolution of the applied noise floor measurements, which was different for EPI and MGRE. However, the current densities (**J**^true^and**J**^rec^) were resampled to the resolution of the MGRE sequence after obtaining the conductivity estimates. The current density error was calculated in all tissue types with the lowest slice in the 5-slice EPI data acting as the lower boundary since the current density and the validation are mostly of interest near the electrodes. No upper boundary was used.

The noise floors from both the EPI and MGRE sequences were added to the simulation. For that, one brain slice was measured without current injection with the MGRE sequence with a total acquisition time of 8:40 min. A time-matched EPI measurement was performed for the same slice. Additionally, to get wider brain coverage, 5 slices were also measured with the EPI sequence where the acquisition time was 1:44 min per slice and 8:40 min in total.

### 2.6 Experiment 3: Optimization of conductivities with injected current measurements

For the third experiment, we performed MRCDI measurements with ±1 mA currents injected in synchrony with the MR sequence using a right-left (RL) and an anterior-posterior (AP) electrode montage. As in the previous experiment, three measurements were performed: one brain slice with the MGRE sequence, one time-matched slice with EPI, and 5 slices with EPI with 1:44 min acquisition time per slice. Only 4 slices were used for subject 2 in the 5-slice EPI data due to acquisition errors for one of the slices, most likely caused by a failed synchronization between the MR sequence and the current source. The measured data${\text{B}}_{\text{z,c}}^{\text{m}}$were used in the optimization of conductivities according toequation 1, where the literature conductivities are the same as used for the previous experiment. In this case, the ground truth is unknown, and δB_z,c_indicates a difference between measured and simulated B_z,c_. The difference was evaluated before (${\text{δB}}_{\text{z,c}}^{\text{lit}}$ ) and after (${\text{δB}}_{\text{z,c}}^{\text{opt}}$ ) optimization, where the literature conductivities were used to calculate the magnetic field before optimization.

Optimizations were performed individually for each subject for separate RL and AP montages as well as for both RL and AP montages together. To avoid overfitting of conductivities to individual subjects, inter-subject optimizations were also performed with leave-one-out cross-validation (LOOCV) where four subjects were used for optimization and${\text{δB}}_{\text{z,c}}^{\text{opt}}$was evaluated for the remaining subject with the obtained conductivity values. RL and AP montages were both used separately and together for the LOOCV optimizations. The LOOCV optimizations were only performed for the 5-slice EPI data.

## 3 Results

### 3.1 Experiment 1: Verifying the EPI sequence with a wire loop

The results from the loop experiment are presented inFigure 2. The residuals reveal that there is a good correspondence between the${\text{δB}}_{\text{z}}^{\text{m}}$calculated from phase difference images and the estimated stray field calculated with Biot-Savart law. However, some remaining residuals still exist, which can have multiple causes, such as errors in the cable tracking or subject movement during scanning. Sulci regions, especially in the upper slices, are also slightly visible, which can be caused by CSF flow.

**Fig. 2.**
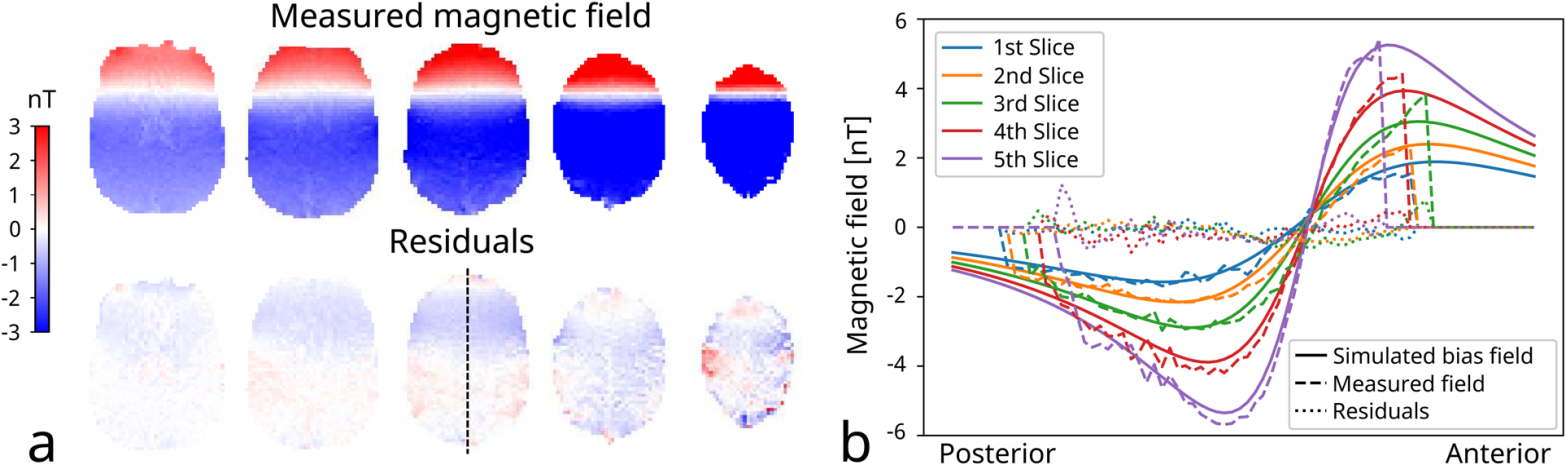
Results from the wire loop experiment. (a) The first row shows the measured magnetic fields while the second row shows the residuals after subtraction of the magnetic field calculated using the Biot-Savart equation. (b) The plot shows a posterior-to-anterior profile through the simulated and measured magnetic fields and the residuals (indicated by the dashed line in the middle residual image). The slices numbers shown in b are ordered from the inferior to the superior position.

### 3.2 Experiment 2: Optimization of conductivities with added noise floors

The noise floor measurements for each subject with the MGRE and 1-slice EPI sequence are presented inFigure 3. Although the noise floor images for EPI and MGRE are not directly comparable due to the different voxel size and different point spread functions, it is clear that the MGRE sequence has stronger low-frequency noise patterns that do not exist in the EPI images.Figure 4shows all the measured magnitude (row 1) and noise floor images (row 3) for one subject as well as the simulated magnetic fields (row 2) with added measurement noise (row 4). The spatial resolution in the simulation is lower for the EPI sequence, which could result in loss of high-frequency structures in the magnetic field resulting in larger errors. However, it is clear from the calculated current density errors presented inFigure 5that the optimization with noise from the EPI data outperforms the optimization with noise from the MGRE data. There is negligible difference between 1-slice EPI and 5-slice EPI acquired over 8:40 min in total.

**Fig. 3.**
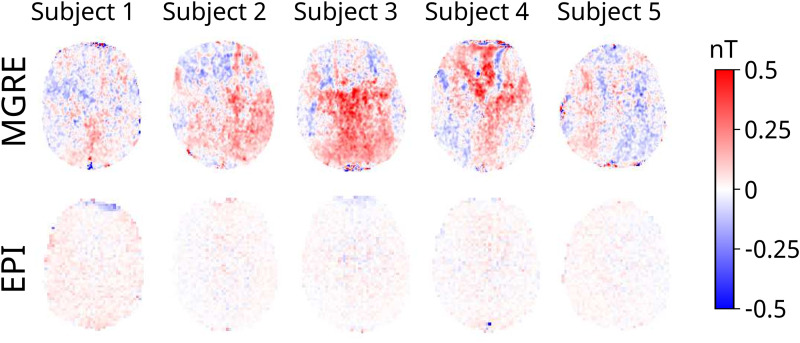
Noise floor measurements for all five subjects for the MGRE and 1-slice EPI acquisitions. The total acquisition time was 8:40 min per measurement.

**Fig. 4.**
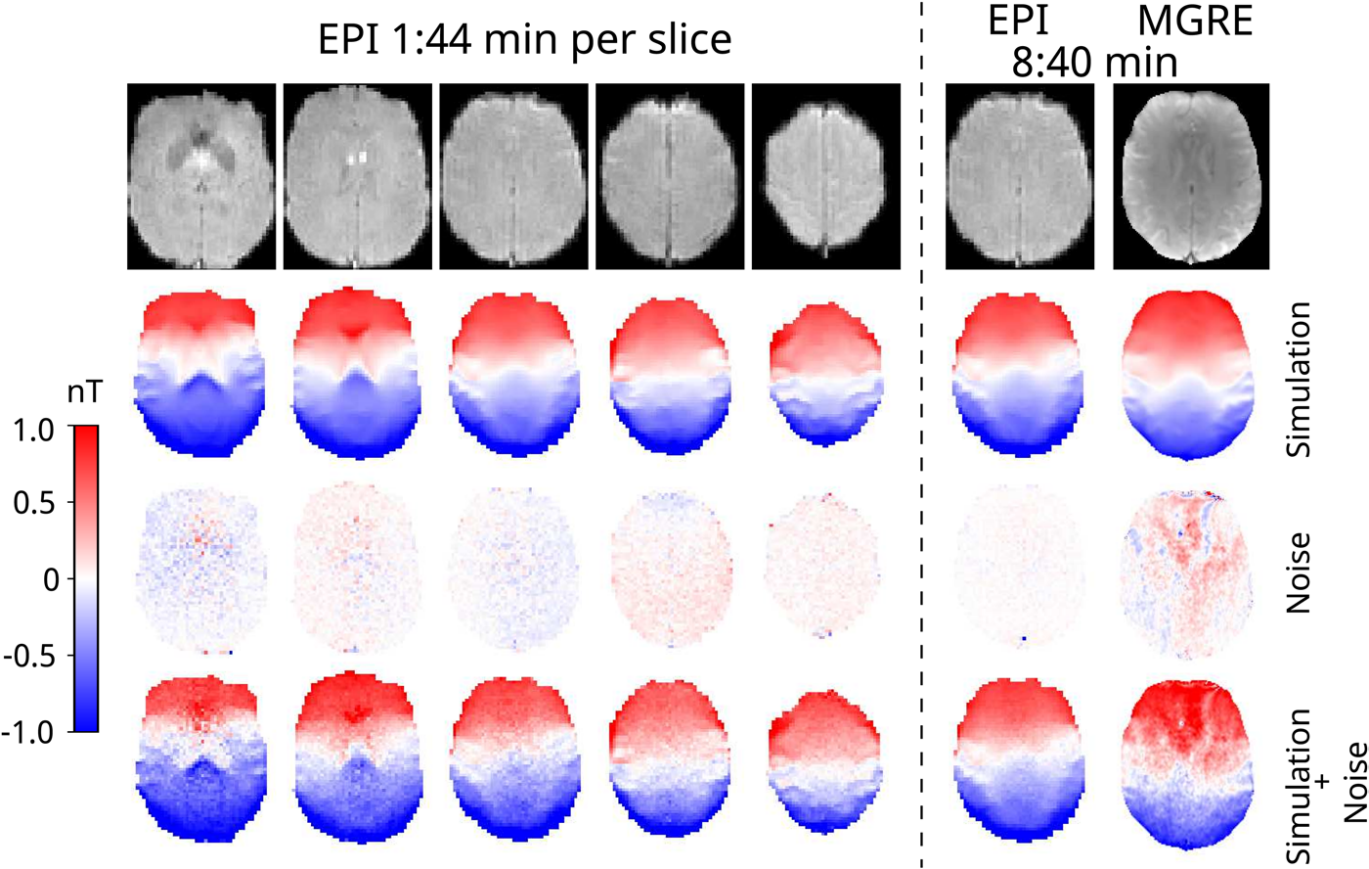
The simulation experiment with added noise from measurements for an example subject. The 5-slice EPI measurements are shown on the left and the 1-slice EPI and MGRE on the right. The top row shows the magnitude images from the respective sequences and slices. The second row shows the simulated magnetic fields with the initial conductivities, while the third row shows the MRCDI measurements without injected currents (noise floor measurements). The fourth row shows the noise floor added to the simulations.

**Fig. 5.**
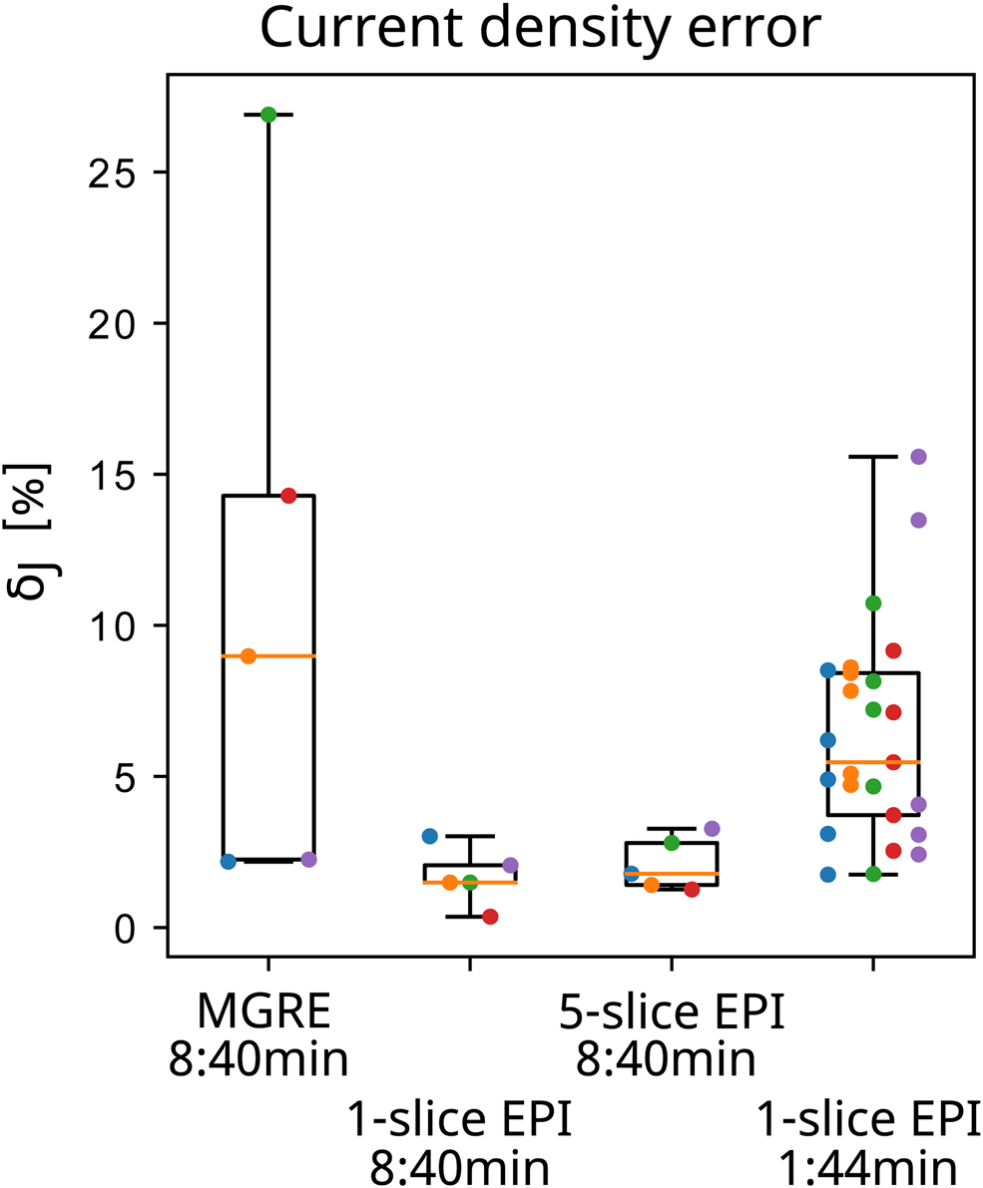
Current density error after optimization with noisy simulated magnetic fields. The 1-slice EPI in the fourth box covers the same slices as used in 5-slice EPI but with optimization done separately for each slice. The horizontal orange lines indicate the medians, the black boxes extend from 25th to 75th percentile, and the whiskers indicate the full range. The colored dots represent each subject.

### 3.3 Experiment 3: Optimization of conductivities from injected current measurements

The data for one example subject is presented inFigure 6with all measured and corresponding simulated magnetic fields. Especially the top slices in AP measurements are extraordinarily similar. The larger differences for the lower slices can for example be caused by expected inaccuracies in the lower part of the head model where the anatomy is more complex. There is visually a larger difference between the measurement and simulations with literature conductivities in the RL electrode montage. After optimization, the similarity is improved substantially. This is also clear inFigure 7, where the quantitative differences are presented in the box plots. Optimizing the conductivities has more influence on the difference in the RL data. There is very little difference between the magnetic field measurements acquired with the MGRE and the EPI sequence, which can be seen visually inFigure 6and quantitatively inFigure 7a.

**Fig. 6.**
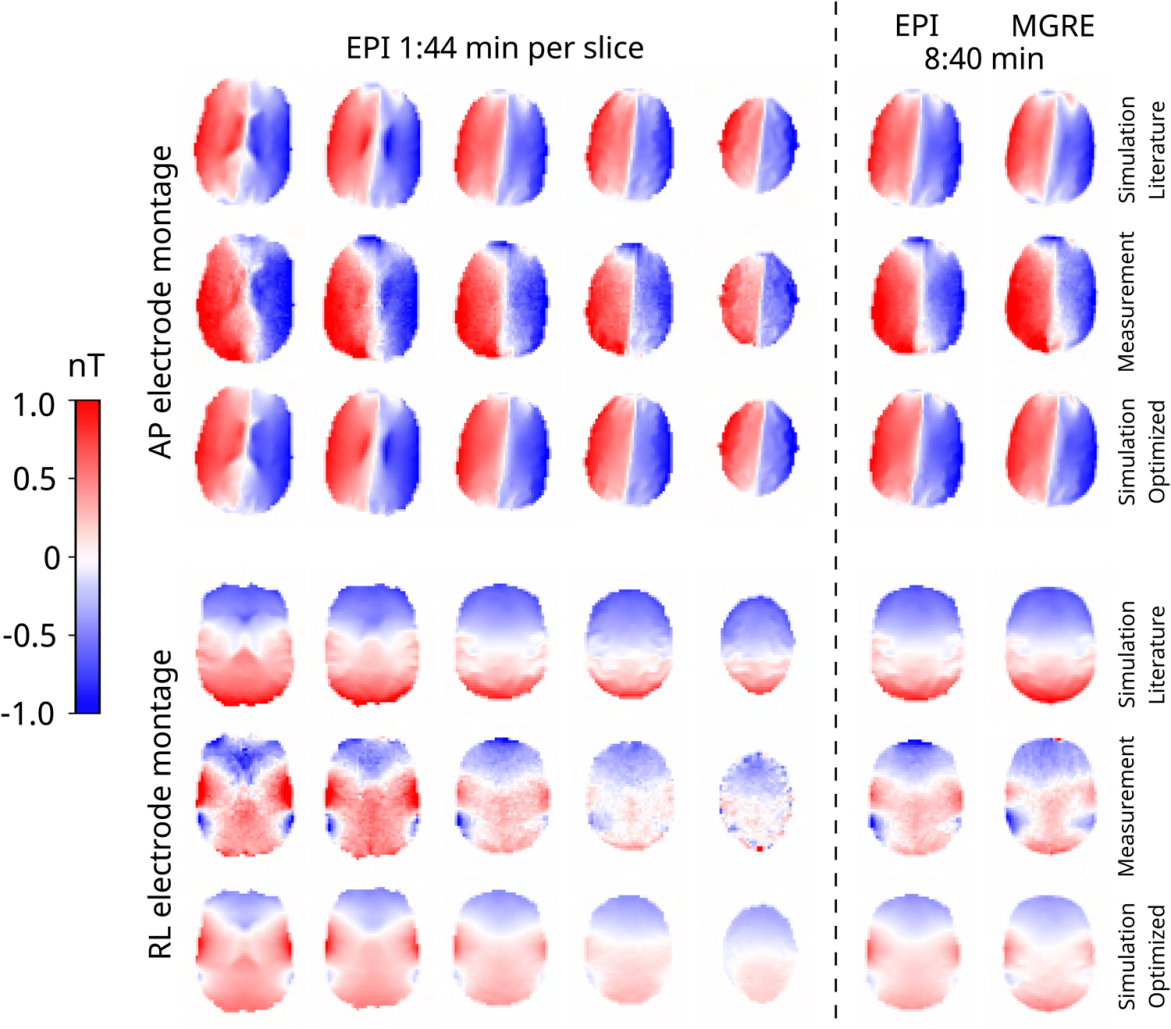
An example dataset for one subject with both the AP (top) and RL (bottom) electrode montages. The first row for each electrode montage shows the magnetic fields simulated with the literature conductivities. The second row shows the measurements, and the third row shows the simulated magnetic fields with the conductivities obtained from the conductivity optimization.

**Fig. 7.**
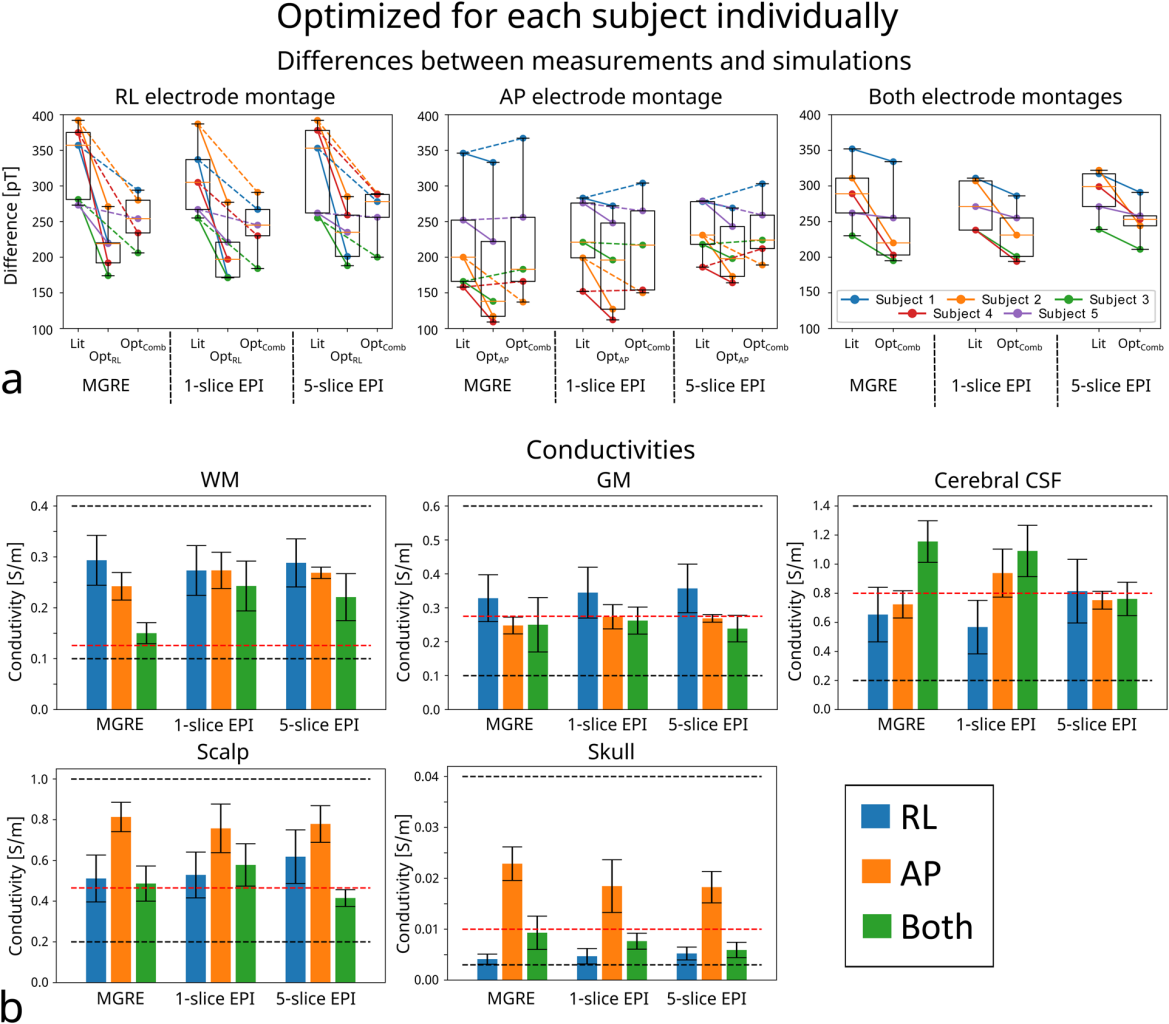
Results from conductivity optimization for individual subjects based on the current injection experiments. (a) The differences between the measured and simulated magnetic fields. The three plots show the differences calculated for the RL, AP, and for both electrode montages, respectively. Each plot displays the error for all three time-matched measurements and for simulated magnetic fields using literature conductivities (Lit), conductivities optimized for one electrode montage (OPT_RL_or OPT_AP_), and conductivities optimized for both montages combined (OPT_Comb_). The horizontal orange lines indicate the medians, the black boxes extend from 25th to 75th percentile, and the whiskers indicate the full range. The lines between the box plots connect the same subject before and after optimization (the full and dashed lines in the two first plots are for the optimization for the single montage and both montages, respectively.) (b) The mean and standard error of the conductivities for all five subjects optimized individually. Optimization was performed for RL and AP individually (blue and orange) as well as combined (green). The black dashed lines indicate the upper and lower bounds used in the optimizations while the red dashed lines are the literature conductivities.

The mean and the standard error of the conductivities after optimization for each subject are presented in the bar plots inFigure 7b. Optimization was performed for RL and AP electrode montages separately as well as for both montages simultaneously. Similar conductivity outcomes are obtained for the three different measurements (MGRE, 1-slice EPI, and 5-slice EPI) with the same electrode montage, further emphasizing that limited differences are observed between measurement techniques. However, the three optimizations (RL, AP, and combined electrode montages) have different outcomes, especially for scalp and skull conductivities.

Results for LOOCV are presented inFigure 8. The conductivity and difference calculations are comparable to the individual subject optimizations presented inFigure 7. The differences are slightly larger, which is expected with inter-subject optimization. However, for the RL montage, all subjects improve in the LOOCV indicating a consistent difference between simulations and measurements for all subjects. The initial difference for the AP electrode montage is considerably smaller. Less improvement is also obtained with conductivity optimizations for the AP compared with RL montage, both for individual subject optimization and for LOOCV. Optimization using both electrode montages with the LOOCV approach also resulted in an improvement for all left-out subjects when evaluated on the RL measurement and both measurements combined, with little difference when evaluated on the AP montage. In summary, the LOOCV results show that the reduction of the error after optimization generalizes well between subjects, indicating that MRCDI can provide a set of conductivities that will in general improve the electric field simulations for new subjects.

**Fig. 8.**
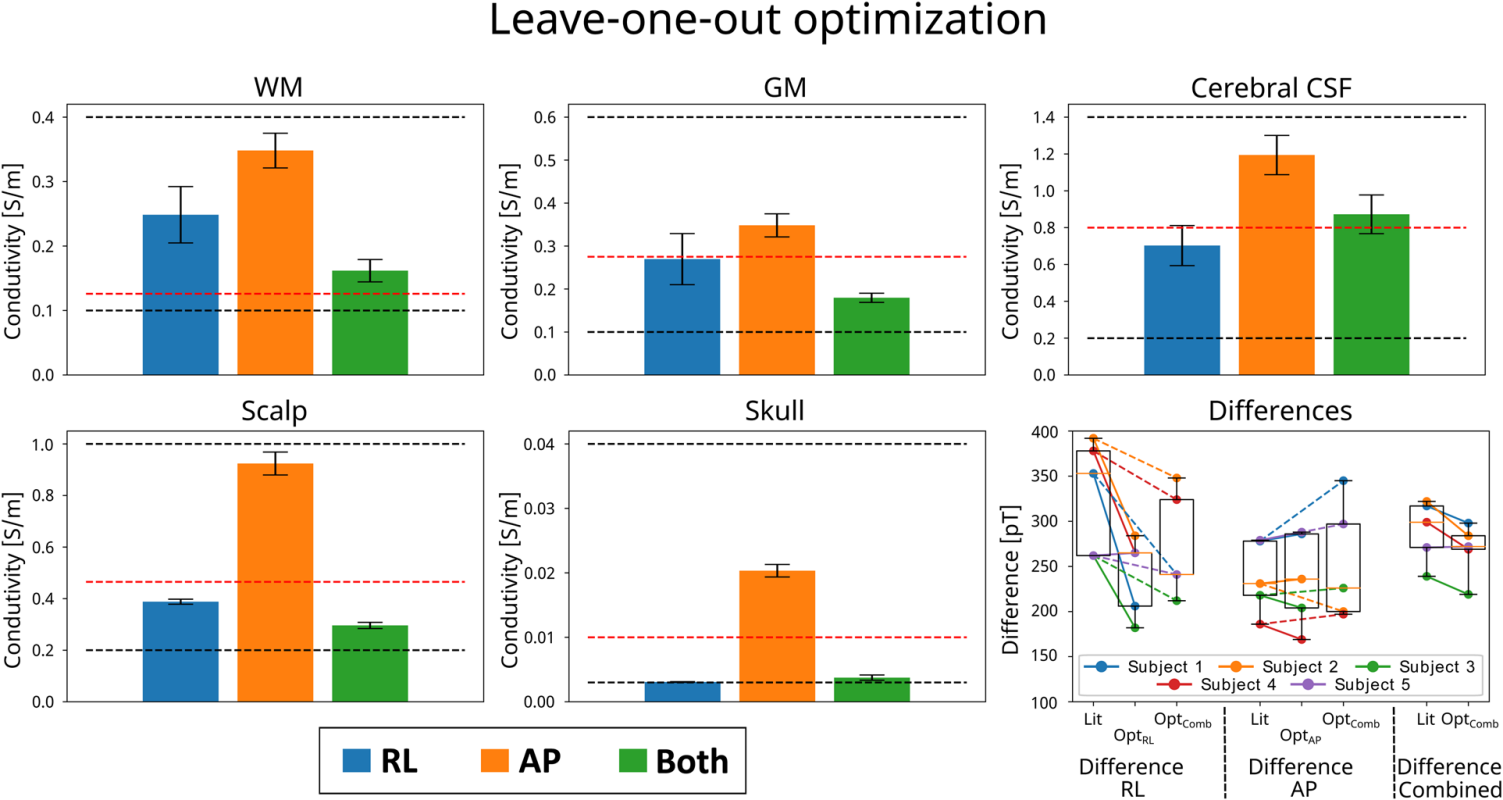
Results from the leave-one-out optimization based on the 5-slice EPI data. Conductivity optimization was performed five times for four subjects with the RL (blue), AP (orange), and both (green) electrode montages with one subject left out. The mean and standard error of the obtained conductivities from the five optimizations are shown above. The black dashed lines are the upper and lower conductivity limits while the red line indicates the initial conductivity value. The difference between measurement and simulations was calculated for the left-out subject (bottom right plot). The differences were calculated for the RL, AP, and both electrode montages, respectively. For each electrode montage, the difference was calculated with literature conductivities (lit), conductivities optimized for one-electrode montage (OPT_RL_or OPT_AP_), and for both electrode montages combined (OPT_Comb_).

## 4 Discussion

With the first validated, reliable human in-vivo EPI-based MRCDI measurements, we have demonstrated that EPI is a good alternative to MGRE, especially if low-frequency noise patterns in the magnetic field measurements are an issue. Due to the rapid sampling of images, physiological noise has less effect on the EPI-based field mapping. However, a well-known problem for EPI is susceptibility-induced signal loss, especially for lower slices close to the paranasal sinuses. This is also a problem for MRCDI, where a long current injection and readout time are necessary. We used a double-echo EPI sequence and combined the echoes based on the variance of each echo calculated from the signal magnitudes for individual voxels. This provides reasonably low noise B_z_estimates, even in relatively low${\text{T}}_{2}^{*}$regions. However, as visible inFigure 4, slightly higher noise is observed in the lowest slices, where${\text{T}}_{2}^{*}$is low. Geometrical distortion is also a well-known issue for EPI acquisitions. The double-echo EPI sequence has a high bandwidth per pixel in the phase encoding direction, resulting in reduced geometrical distortions compared with a single-echo EPI with the same total read-out time. Additionally, we used a lower resolution for the EPI sequence compared with the MGRE sequence. However, since the magnetic fields in the brain caused by injected currents are mainly low frequency, the lower resolution is not a limitation for the method of optimizing conductivities based on the magnetic fields, as used here. While expected, we performed initial simulations of varying resolution for confirmation (data not shown). This notion is also strengthened by our simulation experiments with added noise floors from measurements (Figures 4&5). With the error in the current density estimation being largest for the MGRE sequence, it suggests that reducing the noise level, especially the low-frequency noise, is more important than having a high resolution. However, the performance of a specific optimization algorithm will likely depend on a trade-off between resolution and noise, and this might thus differ for other methods.

We have here used a recently suggested approach (Eroğlu et al., 2021) that optimizes the conductivities of personalized volume conductor models of the head by minimizing the differences between measured and simulated magnetic fields. Consistently in all five subjects (Fig. 7), the simulations agree better with the measurements for the AP montage than for the RL montage, and this difference persists also after optimizing the tissue conductivities. This suggests that the residual unexplained variance might provide valuable insight into segmentation biases or oversimplifications. This might potentially also help to guide a systematic improvement of the segmentation methods, for example by adding tissue types to increase the amount of anatomical detail in the head models (Puonti et al., 2020).

On the other hand, also consistently for all five subjects, optimizing the tissue conductivities improved the simulation accuracy more for the RL than for the AP montage. Interestingly, even a combined optimization to simultaneously fit the measurements for both montages resulted in stable improvements for the RL montage, while the fits for the AP montage on average neither improved nor got worse. Importantly, this points toward the possibility to derive subject-specific tissue conductivities that robustly improve the overall model accuracy when combining measurements of several complementary montages.

The results were consistent for all measurement approaches (MGRE, 1-slice EPI, and 5-slice EPI), demonstrating the robustness of the results. The time-matched 5-slice EPI performs comparably with 1-slice EPI both in the simulation study with added measurement noise (Fig. 5) and for injected currents (Fig. 7) despite higher noise in each slice. Even though there is limited difference between the 1-slice and 5-slice EPI, it is likely still preferable to obtain multiple slices rather than reducing the noise for 1 slice in order to avoid overfitting of the conductivities to a limited region of the head model. Including multiple slices in the optimization can also help with detecting inaccurate regions of the head models.

We extended the optimization of tissue conductivities to group data using leave-one-out cross-validation (Fig. 8). The goal was to see whether a single set of tissue conductivities could be obtained that improved the fit of the simulated fields to the measurements across all subjects. This did not work for the AP montage, suggesting that the literature conductivities are close to optimal in that case. However, a consistent improvement was again seen for the RL montage. While restricted to a proof-of-concept with only five subjects here, this indicates the potential of the approach to systematically derive tissue conductivities that on average improve the simulation accuracy also for unseen (new) participants, given a sufficiently large group dataset. Encouragingly, even when determining a single set of tissue conductivities across all subjects and both montages, the simulation accuracy for the AP montage stayed on average unaffected, while accuracy for the RL montage improved. If replicable in a larger group and additional montages, this would enable us to provide a set of conductivities that generally optimize simulation accuracy and—similarly important—allow us to state uncertainty bounds on the electric field simulations.

### 4.1 Relation to previous work

A previous study using multi- and single-shot spin-echo EPI acquisition in phantoms has been performed (Chauhan, Vidya Shankar, et al., 2018). While currents were only injected for a short period between the excitation and refocusing pulse, we have in this study applied currents throughout the whole readout period, which results in increased efficiency.

Two other previous studies have attempted to measure current-induced magnetic fields with EPI with current injected during readout (Jog et al., 2016,^2020^). However, lead stray field correction was not performed, leading to large differences between simulated and measured magnetic fields. Additionally, with the aim of mapping direct currents constant over a period of minutes, the prior method was not robust to physiological noise or hardware instabilities.

MRCDI has great potential to serve as a safe and non-invasive method to validate and guide the improvement of computational head models. As alternatives to the approach used here and that is based on the optimization of ohmic conductivities of subject-specific head models, methods have been suggested that would in principle enable voxel-wise conductivity reconstructions from the measured${\text{B}}_{\text{z,c}}^{\text{m}}$ . However, they are not ready yet to enable faithful reconstructions of human brain conductivities. Some algorithms directly reconstruct a spatial conductivity distribution from the measured${\text{B}}_{\text{z,c}}^{\text{m}}$ . The most popular algorithm of this type, termed harmonic B_z_algorithm (Oh et al., 2003), requires the computation of the Laplacian of the B_z_, which strongly amplifies the measurement noise. This is problematic for the inherently low SNR of the measured current-induced magnetic field measurements, and successful use of the algorithm to reconstruct human brain conductivity has not been demonstrated so far.

Another class of algorithms reconstructs a spatial conductivity distribution from the current densities (**J**-based) (Woo et al., 1994), and thus require initial estimations of the current densities that caused the measured${\text{B}}_{\text{z,c}}^{\text{m}}$ . However, the latter step is challenging as well. A voxel-wise reconstruction of the full current density vector field would either require knowledge of all three magnetic field components or repeated measurements of the current densities for different object rotations, which are both not feasible for in-vivo human brain MRCDI. Alternatively, voxel-wise reconstructions of two components of the current density from measurements of one magnetic field component have been proposed that work well in phantoms with specific simple geometries (Jeong et al., 2014;Park et al., 2007), but fail in the case of the human brain due to the more complex head anatomy (Eroğlu et al., 2021). In addition, they rely on first-order spatial derivatives of the measured${\text{B}}_{\text{z,c}}^{\text{m}}$ , which poses stringent requirements on the measured SNR (even though less than the computations of the Laplacian of the B_z_in the case of the harmonic B_z_algorithm).

A linear relationship exists between the eigenvalues of the water diffusion tensor and the conductivity tensor (Tuch et al., 2001). MREIT in combination with diffusion tensor imaging (DT-MREIT) has been attempted to determine the position-dependent scaling factor relating diffusivity to conductivity (Kwon et al., 2014) and further used to determine the anisotropic conductivity tensor (Jeong et al., 2017). However,Eroğlu et al. (2021)have in simulation shown that adding anisotropic conductivity to the head model only minimally influences the current-induced magnetic field and can thus not explain the difference between measured and simulated fields observed in this study. Whether determining anisotropic conductivity tensors from the measured magnetic field is sensitive enough has yet to be determined.

Importantly, all voxel-wise reconstruction algorithms give information only for the tissues inside the measured volume or slices. In addition, conductivities cannot be recovered for low-SNR regions such as the scalp and skull, which are therefore masked out in the measurements. Given the low focality of many montages for transcranial electric stimulation and the large sensitivity of the simulated E-fields used in transcranial electric stimulations to skull and scalp conductivities (Saturnino et al., 2019), this significantly limits the practical utility of voxel-wise conductivity reconstructions.

### 4.2 Limitations and future work

The main aim of this work was to assess whether repeated ultra-fast imaging of current-induced fields using EPI would provide reliable results comparable with previously used MGRE sequences while also reducing low-frequency noise. The hypothesis was that faster sampling of MR images would result in less low-frequency noise, which is most likely caused by physiological variations since they are absent in phantom measurements. We also tested time-matched 1-slice and 5-slice EPI acquisitions, which provided comparable results for both the simulation study with added measurement noise and for injected currents. In the future, to leverage the expected benefit of multiple slices, for example, to reduce overfitting to one region of the head model, a reduced total acquisition time or better coverage could be obtained with simultaneous multi-slice (SMS) imaging (Setsompop et al., 2012). However, a systematic approach must be adopted to assess the influence of SMS acquisition on the quality of the B_z_measurements to find an appropriate number of slices and slice distance resulting in minimum added noise and leakage between slices.

We did not perform a direct quantitative comparison of the SNR between the MGRE and EPI sequences. This is challenging since the point spread functions will not be the same. Instead, the performances of the two acquisition methods were compared with respect to the model-based conductivity optimization defining an error metric. The MGRE was kept as in our previous study and the EPI parameters were limited by the need to fit two echoes within one TR to reduce distortions and signal dropouts. For other analysis approaches, for example voxel-wise current density or conductivity reconstruction, the higher resolution obtained with the MGRE sequence might be beneficial. We also tested the recently suggested conductivity optimization method (Eroğlu et al., 2021) on five subjects with all three measurement approaches. Despite the limited number of subjects used in this study, our preliminary results demonstrate the capability of MRCDI to identify differences in the accuracy of the head models for different electrode montages, even for unseen subjects. Testing more subjects and electrode montages was outside the scope of this study as the main focus was on the development and validation of the EPI approach. However, for future studies with more subjects and electrode montages, the possibility to use more complex head models with additional tissue types (Puonti et al., 2020) should be explored. This would likely reduce the error between measurements and simulations, and especially the discrepancy between the conductivity outcomes for different electrode montages. MRCDI would thus play an important role in identifying which tissue types are necessary to add to the head models by providing a more stable conductivity outcome.

We did not use anisotropic brain conductivity derived from diffusion MR data for the head models in this study. In a previous paper (Fig. S2 inEroğlu et al., 2021) we have demonstrated that adding anisotropy to brain tissue has minimal influence on the simulated magnetic field, and certainly much smaller than the errors in this study. This is not to say that diffusion is irrelevant to consider, but rather that inaccuracies in modeling non-brain tissue is likely the main contributor to the errors observed here. The current density is highly influenced by the complex composition and varying conductivities of the scalp, skull, and muscles, where the quality of the MRCDI data is poor. Diffusion measurements were therefore not acquired for this study. However, future larger cohort studies on conductivity optimization, TES dose control, head model improvements, or similar should preferably include diffusion measurements.

## 5 Conclusion

We have shown that EPI-based MRCDI produces reliable measurements of current-induced magnetic fields in the human brain comparable with previously used MGRE sequences while eliminating spatial low-frequency noise that is present in MGRE-based MRCDI. The higher noise floors in MGRE have a negative impact on conductivity optimization where the difference between measured and simulated magnetic fields is minimized. However, we also showed that the difference between simulated and measured magnetic fields is comparable with both EPI- and MGRE-acquired MRCDI data and consistent between subjects, demonstrating the robustness of the MRCDI measurements and its great potential to improve computational head models. Optimization of conductivities improves the fit markedly for all subjects, especially for the RL electrode montage. Importantly, the LOOCV also showed that the results are generalizable between subjects.

## Data Availability

Parts of the methods is publicly available via our open-source software SimNIBS (www.simnibs.org). This includes the methods for building the head volume conductors from structural MR images, forward simulations of the current densities and current-induced magnetic fields, and non-intrusive generalized polynomial chaos expansions for fast re-evaluation of the current-induced magnetic fields in case of changing conductivities. The scripts for reconstructing the magnetic field measurements from the MR data and the conductivity optimization will soon also be available through SimNIBS. Following the ethical approval of the study, the MR data cannot be made publically available.
